# Supplementary material for: Patterns of Intron Gain and Loss in Fungi
Source: PLoS Biol. 2004 Nov 30;2(12):e422. doi: 10.1371/journal.pbio.0020422 (PMC532390; doi:10.1371/journal.pbio.0020422)
Supplement: Table S1 — Also available at http://genes.mit.edu/NielsenEtAl/. (4.3 MB ZIP). [file pbio.0020422.st001.zip › NielsenEtAl/html/498.html]

AN4862.1.NCU03207.1.MG01248.1.FG00562.1


```
 CLUSTAL W (1.82) Multiple Sequence Alignments - Introns Inserted


Sequence 1: NCU03207.1	419 aa
Sequence 2: FG00562.1	419 aa
Sequence 3: MG01248.1	421 aa
Sequence 4: AN4862.1	417 aa
Alignment Length: 423 aa
Number Identitical Residues: 192 aa
Alignment Score (without introns) 9888


MG01248.1 	MVNPDKVFTLEGKALKLDTAEDLDAHIAALKAMDDVEEVRILGNTLGIGACKRLGEVLST
NCU03207.1	MTVSTKVFSLEGKGLKLDTAEDLEAHIAPLRAMDDVEEVRLLGNTLGVGACKLLGEVLAT
FG00562.1 	MASPNKVFSLEGKGLKLDTAEDIEAHIAPLRSQD-VEEVRILGNTLGVGACQRLGEVLAT
AN4862.1  	-MAPPKIFSLEGKGLKLDSAADIEAHIQPLLESTDYTEVRLGGNTLGVPASERLAAVLST
          	   . *:*:****.****:* *::*** .*    .  ***: *****: *.: *. **:*

MG01248.1 	KNNLQ0VANLADIFTGRLLNEIPEALTHLLTQITNLPNLTTVNLNDNAFGINTYKPVADF
NCU03207.1	KKTLR0VANFADIFTGRLLNEIPEALSSLLTSILNLPKLNTINLNDNAFGLNTQAPLVAF
FG00562.1 	KKNLR0IANFADIFTGRLLSEIPDAISSLLTSVLNLPKLTTVNLNDNAFGLNVQAPLVAF
AN4862.1  	QKSLE~VAELADIFTSRLLSEIPDALTFLLNALLEIPTLHTINLSDNAFGANTQKPLVDF
          	::.*. :*::*****.***.***:*:: **. : ::*.* *:**.***** *.  *:. *

MG01248.1 	LSSHVPLQHLYLNNNGLGPEAGMYIADALSELYDKKEQARKEGRTDVPDLETVICGRNRL
NCU03207.1	LAAHVPLQHLYLNNNGLGPHAGILIADALSELHAKKEEARKQGQ-DVPYLETVICGRNRL
FG00562.1 	LSAHVPLQHLYLNNNGMGPHAGILIADALSELHGKKEAARKEGK-EVPDLETVICGRNRL
AN4862.1  	LSRHIPLRHLVLNNNGMGPEAGSNIAKALTELAQRKEEARSGGK-EVPLLESIVCGRNRL
          	*: *:**:** *****:**.**  **.**:**  :** **. *: :** **:::******

MG01248.1 	ENGSMKAWAKAFSKHDK-LKEVKMVQNGIRQEGITHLLTEGLSNTLAIQVLDLQDNTFTI
NCU03207.1	ENGSMTAWAKTFRLHNK-VKQIKMVQNGIRQEGISHLLSEGLNHASELQILDLQDNTFTL
FG00562.1 	ENGSMTAWAKAYSLHNK-IKTVKMVQNGIRQEGVSHLLTEGLSHASELKVLDLQDNTFTV
AN4862.1  	ENGSMAAWAHAYKAHAAGIRSVKMTQNGIRQEGISHLLKEGLSHASALEVLDLQDNTFTI
          	***** ***:::  *  .:: :**.********::***.***.::  :::*********:

MG01248.1 	TGATALAKIAPTWTDLRELGVSDSYLGAKGGRLLSRALAKGKNEKLETLRMQYNDMNART
NCU03207.1	KGARALAKVTPGWTELIELGIGDSLLSAKGGVLLSEALRKGKNKKLEILRLQYNEITAPG
FG00562.1 	TGARALSKVVSNWATIQELGVGDSLLGPKGGILVANALAKGKNNKLEILRLQYNEIPAKG
AN4862.1  	TGSTALAGVVSGWPSLRELGVGDCLLSARGGIKLAQALAKAKNQKVEILRLQYNDISAEG
          	.*: **: :.. *. : ***:.*. *..:**  ::.** *.**:*:* **:***:: *  

MG01248.1 	LKSFADTVKDGLPALKKIEINGNKLSEDDVSIAALQKLLEARKEKAGGDVLIEEEWGVDS
NCU03207.1	IKGLALAAEEALPALKKIELNGNKFTEDDEAIIALQDLLEERKERFGGDVVAEDEWGLDS
FG00562.1 	IQAFATAAKDGLPALKRIEINGNILTEDDESIIALQELLEERKEKFAGDIVNEDEWGVDE
AN4862.1  	VKQFLHAAKTALPALRRIELNGNKFQEEDVNVTELSELLESRKEAHGKDDDPEDMWGIDE
          	:: :  :.: .****::**:*** : *:*  :  *..*** ***  . *   *: **:*.

MG01248.1 	LSDLEDDS-DEEEEEESEEEAETEEEIKKTLDKDEKEAKEPATIKDVASKEVDEITQKLR
NCU03207.1	LSDLEEED-EEEEEEEEEEEEEEVEERAEKLIKEAEAAQEEPVVQ-VKDKEVDELAQKLA
FG00562.1 	LDELEDPDSDAEEEEEEEEEDVEPEERAEKLVKEAEEAQEEPVIP-VKDKEVDELAKKLE
AN4862.1  	LDELESED---EEEEEEEEEEEEEEQKAEKVVKDAEIAENEKVAQ-AQDNDVDKLAEAFG
          	*.:**. .   *****.***    *:  :.: *: : *::  .   . .::**:::: : 

MG01248.1 	QTGI
NCU03207.1	KTEL
FG00562.1 	KTEI
AN4862.1  	KTGI
          	:* :
```
